# Analysis of Final Year Veterinary Students’ Telephone Communication Skills at a Veterinary Teaching Hospital

**DOI:** 10.3390/vetsci5040099

**Published:** 2018-12-03

**Authors:** M. Katie Sheats, Sarah Hammond, April A. Kedrowicz

**Affiliations:** College of Veterinary Medicine, North Carolina State University, Raleigh 27606, NC, USA; mkpeed@ncsu.edu (M.K.S.); slhammon@ncsu.edu (S.H.)

**Keywords:** telephone communication, clinical communication, relationship centered care, veterinary education, telephone medicine, relationship-centered care, veterinarian–client–patient communication, communication research methods

## Abstract

Client communication is a core clinical skill that is taught as part of the required curriculum at many veterinary colleges. Although much client communication occurs face-to-face, telephone communication is used to provide patient updates, relay results of diagnostic tests, and check on discharged patients. This research explored fourth year veterinary medical students’ telephone communication skills. We recorded and analyzed the transcripts of 25 calls students made to clients of three different services in the Veterinary Teaching Hospital. Additionally, we explored the perspectives of veterinary educators by distributing a survey to university faculty and house officers (*n* = 57). Results indicate that students excelled at identifying the patient and purpose of the call and incorporating professional language and clear explanations. They require development in providing structure and incorporating core communication skills. Compared with our survey results, the student findings are at odds with clinicians’ expectations of students’ communication abilities. We conclude that additional training is required to familiarize students with expectations regarding telephone communication, including reviewing the case thoroughly, preparing to answer questions and provide explanations, following organizational protocol, and incorporating open ended questions, reflective listening, and empathy. This data will inform design, and help to measure the impact, of telephone communication education and training that will be incorporated into the existing veterinary communication curriculum.

## 1. Introduction

It is well accepted within the medical community (i.e., physician, veterinary, nursing, etc.) that communication is a core clinical competency and an essential component of education for students in healthcare fields [1,2,3,4]. Learning “best practices” in communication is a benefit to both patients and healthcare professionals [5,6,7,8], and in veterinary medicine it is also a benefit to the client [9,10,11]. The literature on communication in physician, nursing and pharmacy training is extensive [8,12,13,14,15,16,17,18,19,20,21,22,23,24,25,26,27], but less research has been conducted on communication in veterinary student training. Despite this limitation, extrapolation from other medical professions has allowed progress in establishing best practices for face-to-face interviews between veterinarians and their clients [28,29,30,31,32]. Specific communication models adapted from human medicine include the Calgary-Cambridge Observation Guide, “relationship-centered care” and the “four core communication skills of highly effective practitioners”, which include: (1) asking open-ended questions, (2) expressing empathy, (3) using reflective listening, and (4) awareness of nonverbal communication [3,29,32].

One expanding platform for healthcare communication is the telephone. In human medicine, telephone communication has become an integral part of clinical practice, with telephone calls accounting for one fifth of physician-patient contacts in fields including general practice, general internal medicine, pediatrics, and obstetrics/gynecology [33]. Public satisfaction with medical telephone consultation is high, with patients identifying less waiting, reduced travel time, and the possibility of increased frequency of contact as benefits [34]. Medical professionals, however, have reported concerns regarding telephone communication, including an inability to use touch as a communication aid, formality, and relative anonymity [34]. Recognizing the importance of telephone communication in patient care and understanding the unique challenges this mode of communication presents, nursing, pharmacy, and medical schools have designed curricula to teach various aspects of telephone communication [14,21,35,36,37]. Specific skills recommended for telephone communication with healthcare clients include: active listening; frequent paraphrasing to ensure the message sent by one party is the message received by the other party; awareness of paralanguage, including pace of speech, pauses, and voice intonation; and offering opportunities to ask questions [34].

From these reported findings, it is clear that healthcare professionals encounter unique challenges when counseling patients and patient caregivers (i.e., veterinary clients) over the phone, and that this form of communication deserves deliberate attention in the veterinary education curriculum. Unfortunately, very little research has focused on telephone communication in veterinary medicine [38,39]. In 2010, Cary et al. [39] reported on integration of a telephone communication training exercise into the junior surgery lab at Washington State University; and, in 2016, Grevemeyer et al. published a framework for vertical implementation of telephone communication skills training for third year veterinary students at Ross University [38,39]. Both of these exercises were designed to use veterinary staff or faculty as simulated clients who participated in one or more clinical scenarios and provided structured feedback to students. Results reported by Cary et al. [39] indicated that veterinary students valued client-telephone communication exercises as part of their junior surgery lab and, in open-ended responses, revealed that they experienced fear about making telephone calls and felt challenged by the amount of time required to prepare for discussions with clients. In the study by Grevemeyer et al., [38] simulated clients reported that veterinary students were most effective at communication skills relevant to the introduction phase of the telephone interview and were least effective at using open-ended questions, funneling, using lay terminology, and closing the interview. Similar to findings in medical education, these authors concluded that telephone communication skills do not naturally develop on the job and require specific training.

At present, North Carolina State University’s (NSCU) College of Veterinary Medicine (CVM) uses simulated clients for veterinary student communication skills training, but only for face-to-face interviews. There are currently no experiential learning exercises for telephone communication in the three-year communication curriculum at NC State. Prior to designing training exercises for telephone communication, we wanted to gain a better understanding of the current level of telephone communication competence of students who have completed our current curriculum. Therefore, the goal of this study was to analyze authentic, recorded telephone calls between 4th year veterinary students and clients of our veterinary teaching hospital to determine which “basic” communication strategies taught in our 1st through 3rd year curriculum are being used for telephone communication during the 4th clinical year. We complemented this data with a voluntary hospital-wide clinician survey, asking faculty, residents, and interns questions about what communication skills they think students use during telephone communication with clients, how they instruct their students regarding telephone communication, and what they think students should learn from client telephone communication. Our overall goal for this project is to use this data to inform design, and measure impact, of telephone specific education and training that will be incorporated into our existing veterinary communications curriculum.

## 2. Materials and Methods

### 2.1. Design

The study design was exploratory and descriptive with a mixed-methods approach. Findings from the initial quantitative analysis of 25 student-client audio recordings were used to inform design of a veterinary educator survey for secondary quantitative and qualitative analysis. In the present study, the secondary analysis helps to inform the initial set of quantitative data. 

#### Current North Carolina State University (NCSU) Communication Training Curriculum

In 2018, the AAVMC (American Association of Veterinary Medical Colleges) introduced a new Competency Based Veterinary Education (CBVE) program that outlines nine domains of competence for veterinary graduates [40]. Each competence domain is composed of competencies and suggested subcompetencies. The 5th Domain of Competence in this framework is communication, and the competencies within that domain are: [5.1] listens attentively and communicates professionally and [5.2] adapts communication style to colleagues and clients [40]. NCSU students are currently exposed to a robust communication curriculum that spans four courses across the first three years of the Doctor of Veterinary Medicine, (DVM) program. In total, students receive 55 hours of classroom instruction in communication, participate in four simulated client interactions with detailed feedback from a communication coach, and engage in peer feedback and self-reflection. Communication instruction includes information and practice with face-to-face client interactions, team communication and collaboration, and written communication. Within this didactic and experiential curriculum, students learn how to structure a client encounter, build client relationships, and incorporate core communication skills, all of which are transferable to telephone interactions. 

### 2.2. Data Collection

#### 2.2.1. Authentic Student-Client Telephone Communication

All NCSU fourth year veterinary students enrolled in equine medicine, small animal internal medicine, and small animal orthopedic surgery clinical rotations were invited to participate in the study during a one-year period. Students on rotation from other accredited veterinary colleges were excluded from the study group. One designated telephone in each clinical service area was equipped to digitally capture all audio recordings. Student use of the designated telephone was completely voluntary. Client consent for audio recordings of telephone conversations is a routine question on the admitting paperwork for the veterinary teaching hospital. 

Recorded calls were stored as audio files in an online call database, organized by clinical service area. Access to the database was password protected and limited to the study’s principal investigators and research staff. From the database of recordings, calls were randomly selected by transcribing every 7th call in the list. Transcribers screened selected calls to ensure that calls by the same student had not been previously transcribed, and that the call had at least three conversational “exchanges” between the student and the client. If the randomly selected call failed to pass quality control measures, the next call was selected as a replacement. Of the 25 students randomly selected, 13 were on orthopedic surgery rotation, 6 were on equine medicine rotation, and 6 were on internal medicine rotation. Transcription was completed by research assistants. 

A standardized rubric (see Appendix A) was used to analyze transcribed calls for four different themes of veterinary-client communication: (1) students incorporating appropriate identification, (2) students providing call structure, (3) students incorporating core skills, and (4) students communicating professionally. Within these four themes, 16 elements were noted as missing/no or complete/yes, with an ‘optimal’ performance score of 16/16. Calls were coded by 2 different evaluators. Inter-rater reliability was calculated for 72 percent of the sample and showed moderate agreement between coders, k = 0.75 as assessed using Cohen’s Kappa [41]. The coders reconciled any discrepancies collaboratively.

#### 2.2.2. Educator Questionnaire

All clinical educators of the Veterinary Teaching Hospital, including senior faculty, interns and residents, were invited via email to complete an anonymous online questionnaire created with Google Forms (see Appendix B). The questionnaire included 19 yes/no questions that addressed educator expectations for student telephone communication strategies (15 items) and training (4 items), as well as two open-ended questions that addressed preparation and learning outcomes for telephone communication experiences on clinical rotations. Respondents were asked to indicate their veterinary career stage as faculty, intern, or resident. Of the clinical educators surveyed, 35 were faculty, 8 were interns, and 14 served as residents in a teaching hospital. 

### 2.3. Data Analysis

Data were analyzed using both quantitative and qualitative methods. The quantitative survey data was analyzed using SPSS, version 25 IBM Corp., Armonk, NY, USA) where Chi-square tests were performed to identify statistically significant differences between groups. Graphpad Prism 7 version (Graphpad Software, LaJolla, CA, USA) was used to calculate and compare the mean (± SE) rubric score of student telephone calls grouped by time of year, using an unpaired, two-tailed student’s *t* test with Welch’s correction. The qualitative survey data was analyzed using NVivo, version 10, a qualitative software package (QSR International, Melborne, Australia). The two open-ended question responses were coded in two phases. The first was inductive, using open and axial coding to gather emerging trends in the data. The second was a deductive coding process in which responses were coded in comparison to the developed standardized rubric [42].

### 2.4. Ethical Considerations

The study was approved by the North Carolina State University Institutional Review Board (No. 6589).

## 3. Results

### 3.1. Analysis of Authentic, Recorded Student-Client Telephone Communications

**Figure 1:**
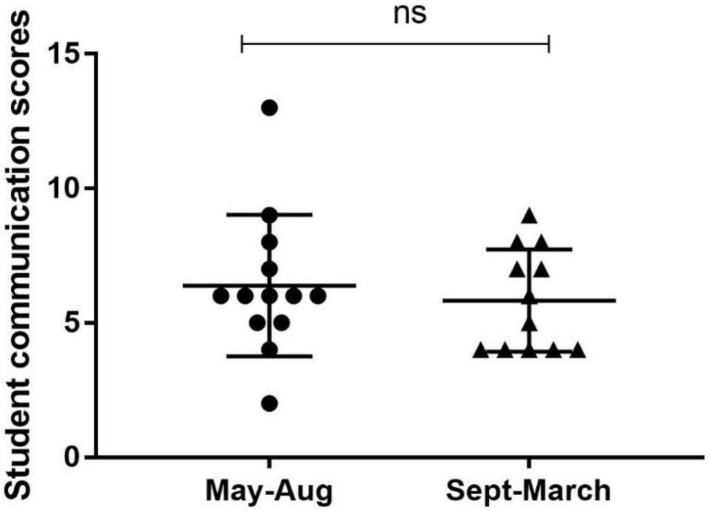
Comparison of mean ± SE rubric score by time of year. Data was analyzed using an unpaired, two-tailed student’s *t* test with Welch’s correction (*p* = 0.5521). (ns = no significant difference).

Table 1 includes a complete breakdown of student performance. First, students were not adept at appropriate identification during telephone conversations. Only 3 students (12%) identified themselves by their full name and identified their role as a student within the hospital to clients. At the beginning of the calls, 14 students (56%) identified the recipient by name, whereas 22 students (88%) identified the patient. With regard to students communicating professionally, only nine students (36%) provided clear explanations to clients. Despite the fact that a majority of students (*n* = 17; 68%) used professional language, nine students (36%) also engaged in unprofessional behaviors such as laughing at inappropriate points in the conversation. Providing structure to the conversation is an important telephone communication skill. This includes explaining the purpose of the call, previewing topics, summarizing, and repeating instructions. Most students (*n* = 20; 80%) explained the purpose of the call, but not one student provided a preview to the topics that would be discussed. Only 13 students (52%) summarized or reiterated next steps, and just nine students (36%) repeated instructions for the client in closing. Students’ also require development at incorporating core communication skills. Only two students (8%) asked an open-ended question, nine (36%) practiced reflective listening, and three (12%) were able to communicate without incorporating vocal segregates such as “um”. Despite having opportunities to communicate empathy in all 25 phone calls, only five students (20%) included an empathetic statement. The rubric used to code student calls consisted of 16 elements, giving an “optimal” communication score maximum of 16. Calls were divided into 2 groups by time of year (May–August vs. September–March), based on the clinical year calendar of May–April. While we speculated that student telephone communication rubric scores would be higher for students with more clinic experience, there was no statistically significant difference between the average rubric score of the two groups (*p* = 0.5521) (see Figure 1). 

### 3.2. Quantitative and Qualitative Analysis of Veterinary Educator Survey Responses 

For quantitative analysis, survey responses were analyzed using Chi-square tests to examine the differences between students’ telephone communication skills and clinician educators’ expectations/perceptions of students’ skills (see Table 1). Statistically significant differences were found for identifying self by full name (χ^2^(1) = 20.694, *p* < 0.001), identifying their role at the hospital (χ^2^(1) = 41.143, *p* < 0.001), providing a preview of the conversation (χ^2^(1) = 4.762, *p* = 0.032), providing clear explanations (χ^2^(1) = 5.173, *p* = 0.022), displaying unprofessional behaviors (χ^2^(1) = 18.137, *p* < 0.001), using vocal segregates (χ^2^(1) = 12.572, *p* < 0.001), and empathizing with the client (χ^2^(1) = 19.133, *p* < 0.001). These findings illustrate the gap that exists between how clinical educators think students should/are communicating with clients while on clinical rotation and what telephone communication skills students are actually incorporating (see Table 1 for detail). 

Of the clinical educators surveyed, almost all of them (*n* = 55, 97%) felt that student-client telephone interactions are an extension of the veterinary care offered by the veterinary teaching hospital and that students should further develop their communication skills as part of the client interactions. Based on qualitative analysis of open-ended survey responses, clinical educators see participation on rotations and communicating with clients over the telephone as opportunities to learn how to communicate complex information and enhance their core communication skills. Despite this finding, only 35 clinical educators (61%) responded that they provide specific guidelines to students for how to communicate with clients over the phone. When asked how they advise students to prepare for calling clients, the most frequent open-ended responses among clinical educators were: to review the case before calling, check on the up to date status of the patient, and anticipate questions the client may have. They also encourage students to seek help from a clinical educator if they do not know something. The feedback students receive with respect to their telephone communication skills varies with 24 clinician educators (42%) indicating that they listen to student phone calls and provide feedback. Clinical educators prefer to serve as examples with the majority of participants (*n* = 52, 91%), indicating that they allow their students to listen to their conversations with clients over the phone. 

## 4. Discussion

The primary goal of this project was to determine the level of communication competency of final year veterinary students during authentic telephone conversations with clients. Students at our university receive three years of didactic and experiential communication training prior to entering their final clinical year; therefore, we hypothesized that students’ telephone communication would benefit from this training and would, at minimum, include elements of core communication skills relevant for telephone communication (open-ended questions, reflective listening, empathy statements). However, our results do not support this hypothesis. While most students identified the patient by name and explained the purpose of the call to the client, only a few students identified themselves and their role, provided a preview of the call, or incorporated core communication skills such as reflective listening, open-ended questions, and empathy. We speculate that these communication deficiencies could be due to lack of a structured approach and dedicated practice, anxiety around telephone communication, and/or lack of attention to preparation and planning. This position is supported by previous work by Grevemeyer et al., who report that veterinary students felt fearful of, and had difficulty preparing for, telephone conversations with simulated clients [38]. It is also possible that trying to accomplish multiple tasks at once (taking ownership of a patient case, organizing medical knowledge, processing diagnostic test results, and communicating with a client) increased students’ cognitive load [43], which adversely impacted their ability to communicate competently. 

When presented with our preliminary findings, some clinical faculty within our veterinary teaching hospital were concerned that “routine phone updates” may not be an adequate way to assess student communication, as some core communication skills (i.e., reflective listening, empathy statements) could be deemed unnecessary in this context. In other words, some clinicians may view telephone communication about routine updates, prescription questions, or discharge follow up as not requiring “best practices” in client communication the way more complex conversations do. While we concede that most of the student calls in our study did not deal with significant conflicts or high-stakes decision-making, we would hope to convince veterinary educators and students alike that every client telephone call is an extension of veterinary healthcare services that could be improved with effective, thoughtful, and purposeful communication. 

Findings suggest that telephone skills used by students in the first half of the clinical year vs. the second half of the clinical year do not change significantly. For veterinary educators this is somewhat disheartening, since general expectations are that 4th year DVM students who are about to graduate should be performing at a higher level, both cognitively and technically, than students at the beginning of their clinical year. However, it is hardly surprising, since previous evidence clearly indicates that in the realm of medical communication, “experience alone is a poor teacher” [44]. Because clinical year students at NCSU are not required to receive feedback or coaching on their telephone communication skills, and only 42% of clinician educators do so voluntarily, students are clearly in need of formalized training, practice, and coaching before expectations for improvement over the course of clinical training will be realized. While it is also important to note that additional research would be needed to determine whether individual veterinary students’ telephone communication skills improve over the course of their clinical year, we plan to focus future education and research efforts on formalized coaching and assessments. 

To further inform our understanding of this data and to gain the perspective of veterinary educators on student training in telephone communication, we surveyed veterinary faculty and house officers. Results from this survey indicate that clinicians see student-client communication as an extension of veterinary care, feel students can learn from telephone conversations with clients, and generally have higher expectations for student telephone communication competency than our recorded data analysis indicates. From these findings, we conclude that additional training is required to familiarize students with expectations regarding telephone communication, including reviewing the case thoroughly, preparing to answer questions and provide explanations, following organizational protocol, and incorporating chunk and check, open-ended questions, reflective listening, and empathy. Moving forward, this data will inform design, and help to measure impact, of telephone specific education and training that will be incorporated into our existing veterinary communications curriculum.

## 5. Conclusions

In summary, this research points to further opportunities to develop students’ telephone communication skills. We recommend developing clear expectations regarding telephone communication including thoroughly reviewing the case, preparing to answer questions and provide clear explanations, following organizational protocol, and incorporating chunk and check, open-ended questions, reflective listening, and empathy. We also recommend experiential training during the clinical year to facilitate development of students’ telephone communication skills, including coaching them in preparation of making calls so they will be able to provide informed updates and instructions and anticipating client questions so they are able to communicate in a way that clients will deem valuable. Finally, we recommend recording student phone conversations and providing them with specific, detailed feedback regarding these interactions.

## Figures and Tables

**Table 1:** Student coding v. clinical educator survey responses.

Communication Categories	Students				Clinical Educators				
	Yes		No		Yes		No		
	*n*	%	n	*%*	*n*	%	n	*%*	*p*-Value
Identify self by name	3	12.0	22	88.0	38	66.7	19	33.3	0.000 *
Identify role	3	12.0	22	88.0	52	91.2	5	8.8	0.000 *
Identify recipient	14	56.0	11	44.0	37	64.9	20	35.1	0.300
Identify patient	22	88.0	3	12.0	27	100.0	0	0	0.026 ^†^
Explain purpose of call	20	80.0	5	20.0	53	93.0	4	7.0	0.092
Provide preview	25	100.0	0	0	13	22.8	44	77.2	0.005 ^†^
Clear explanations	9	36.0	16	64.0	41	71.9	16	28.1	0.002 *
Professional language	17	68.0	8	32.0	40	70.2	17	29.8	0.520
Unprofessional behaviors	9	36.0	16	64.0	51	89.5	6	10.5	0.000 *
Vocal segregates	22	88.0	3	12.0	23	40.4	34	59.6	0.000 *
Reflective listening	9	36.0	16	64.0	26	45.6	31	54.4	0.286
Open-ended questions	2	8.0	23	92.0	30	52.6	27	47.4	0.000 *
Summarize next steps	13	52.0	12	48.0	28	49.1	29	50.9	0.500
Repeat instructions	9	36.0	16	64.0	26	45.6	31	54.4	0.286
Use empathy statements	5	20.0	20	80.0	46	80.7	11	19.3	0.000 *

* Indicates statistically significant results. ^†^ While chi-square results are statistically significant, expected cell count is less than 5.

**Figure A1:**
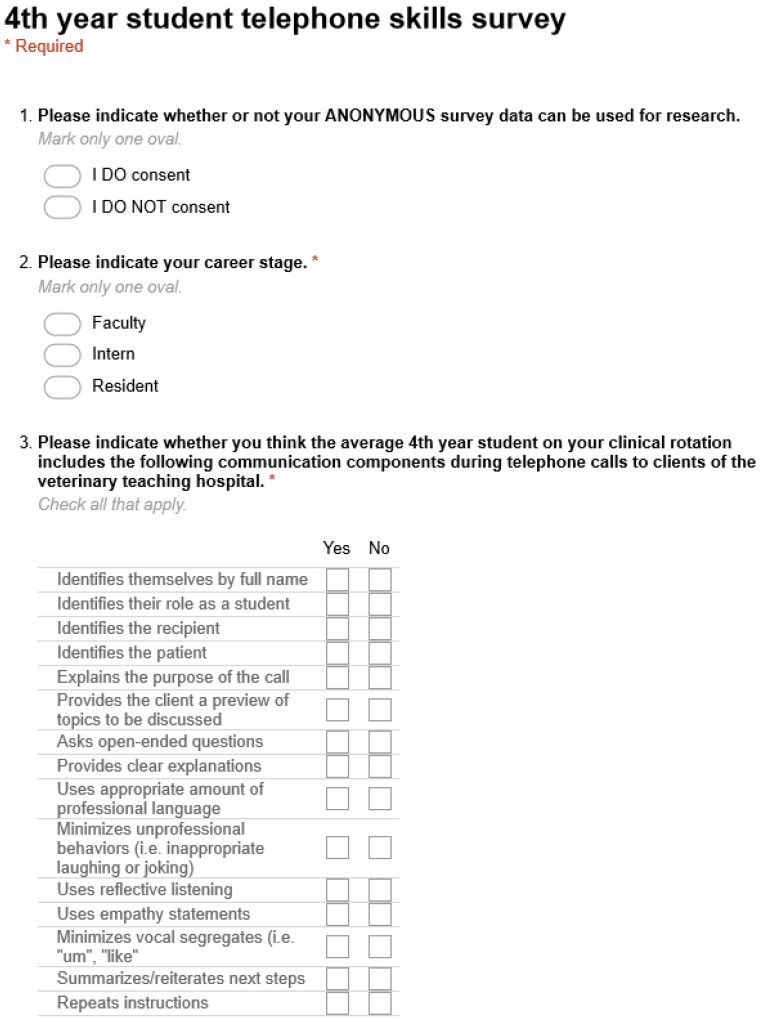

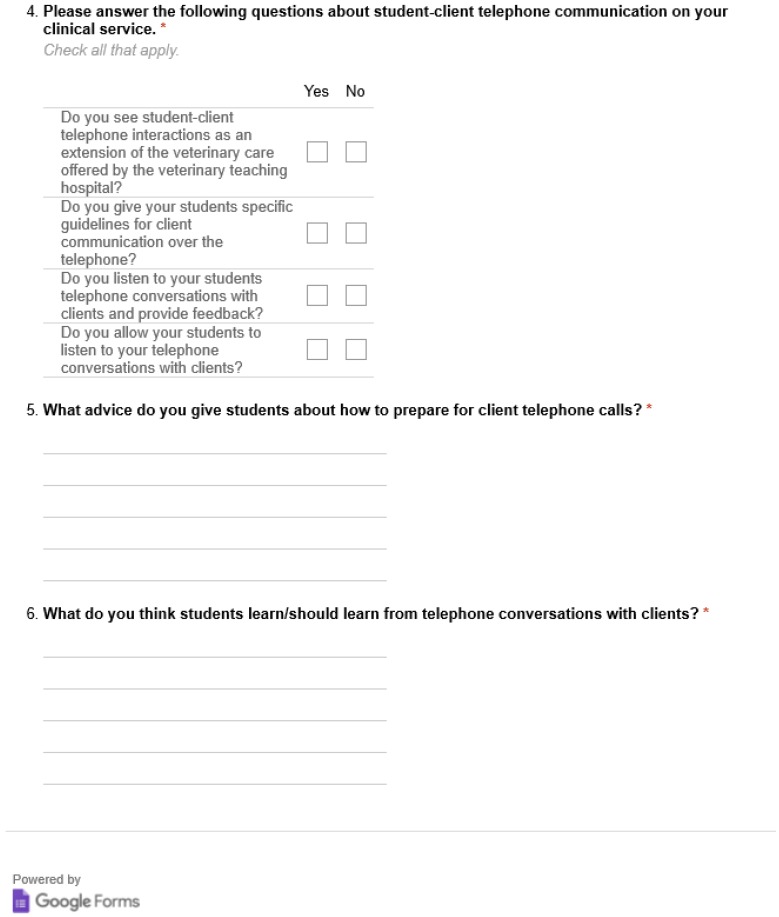
The anonymous online questionnaire.

## Appendix A

**Table A1:** Telephone Communication Coding Scheme.

Student:	Service:	Call Length:
Missing/No	Item to Be Coded	Completed/Yes
	Identify self by full name	
	Identify role	
	Identify recipient	
	Identify patient	
	Explains purpose of call	
	Provides preview	
	Clear explanations	
	Professional language	
	Unprofessional behaviors (Laughing)	
	Vocal segregates (ums)	
	Reflective listening	
	Interruptions	
	Empathy	
	Open-ended questions	
	Summarize/reiterate next steps	
	Repeat instructions	
